# Repeated measures study of weekly and daily cytomegalovirus shedding patterns in saliva and urine of healthy cytomegalovirus-seropositive children

**DOI:** 10.1186/s12879-014-0569-1

**Published:** 2014-11-13

**Authors:** Michael J Cannon, Jennifer D Stowell, Rebekah Clark, Philip R Dollard, Delaney Johnson, Karen Mask, Cynthia Stover, Karen Wu, Minal Amin, Will Hendley, Jing Guo, D Scott Schmid, Sheila C Dollard

**Affiliations:** National Center on Birth Defects and Developmental Disabilities, Centers for Disease Control and Prevention, 1600 Clifton Road, Mailstop E-86, Atlanta, 30333 GA USA; Emory University, Atlanta, GA USA; National Center for Immunization and Respiratory Diseases, Centers for Disease Control and Prevention, Atlanta, GA USA

**Keywords:** Cytomegalovirus, Congenital, Awareness, Behavior, Pregnancy

## Abstract

**Background:**

To better understand potential transmission risks from contact with the body fluids of children, we monitored the presence and amount of CMV shedding over time in healthy CMV-seropositive children.

**Methods:**

Through screening we identified 36 children from the Atlanta, Georgia area who were CMV-seropositive, including 23 who were shedding CMV at the time of screening. Each child received 12 weekly in-home visits at which field workers collected saliva and urine. During the final two weeks, parents also collected saliva and urine daily.

**Results:**

Prevalence of shedding was highly correlated with initial shedding status: children shedding at the screening visit had CMV DNA in 84% of follow-up saliva specimens (455/543) and 28% of follow-up urine specimens (151/539); those not shedding at the screening visit had CMV DNA in 16% of follow-up saliva specimens (47/303) and 5% of follow-up urine specimens (16/305). Among positive specimens we found median viral loads of 82,900 copies/mL in saliva and 34,730 copies/mL in urine (P = 0.01), while the viral load for the 75th percentile was nearly 1.5 million copies/mL for saliva compared to 86,800 copies/mL for urine. Younger age was significantly associated with higher viral loads, especially for saliva (P < 0.001). Shedding prevalence and viral loads were relatively stable over time. All children who were shedding at the screening visit were still shedding at least some days during weeks 11 and 12, and median and mean viral loads did not change substantially over time.

**Conclusions:**

Healthy CMV-seropositive children can shed CMV for months at high, relatively stable levels. These data suggest that behavioral prevention messages need to address transmission via both saliva and urine, but also need to be informed by the potentially higher risks posed by saliva and by exposures to younger children.

**Electronic supplementary material:**

The online version of this article (doi:10.1186/s12879-014-0569-1) contains supplementary material, which is available to authorized users.

## Background

Congenital cytomegalovirus (CMV) infection is an important cause of birth defects and developmental disabilities, including hearing loss, vision loss, and intellectual disability [1],[2]. CMV infection can be transmitted to the fetus during pregnancy when the mother has a primary (i.e., first) infection, a re-infection with a new strain, or a viral reactivation [3]. Preventing primary or re-infection through behavioral changes [4]-[6] is of great importance, especially since there is no licensed vaccine [7],[8] or established treatment for pregnant women [9]-[11].

In order to more successfully prevent CMV transmission to pregnant women, it is important to improve our understanding of transmission modes. CMV can be transmitted in several ways, all of which require close contact with infected body fluids. Although transmission through intimate contact (e.g., kissing, sex) with an infected adult is important [12],[13], especially among those with multiple sexual partners, seroconversion studies suggest that, for many women, exposure to young children may be even more important [14].

To better understand the potential risk associated with exposure to saliva and urine of healthy CMV-seropositive children, we carried out a repeated measures study of CMV shedding (i.e., presence of viral DNA in body fluids). In contrast to most previous studies, we measured viral loads, tested both saliva and urine, and assessed changes over time, including at weekly and daily intervals. Through this study we hoped to inform and refine our knowledge about transmission modes in order to better inform behavioral prevention messages for pregnant women.

## Methods

### Study population

We identified participants for longitudinal follow-up from a convenience sample of children without chronic medical conditions aged 0-47 months in the Atlanta, Georgia metropolitan area (Stowell et al., companion paper). None of the children had a diagnosis of congenital CMV infection. To be eligible for follow-up, the children first had to test positive for anti-CMV IgG-50 of 161 children met this criterion (Stowell et al., companion paper). Of these children, we enrolled 23 of 25 (92%) who also tested positive for CMV DNA in saliva and/or urine. In addition, we enrolled some seropositive non-shedders (N = 13) so that we could assess spontaneous initiation of shedding. We obtained approval for the study from the Institutional Review Board (IRB) at the U.S. Centers for Disease Control and Prevention (CDC), and we obtained informed consent for the children from their mothers.

### Specimen collection and storage

After the initial screening visit, we carried out 12 in-home visits for each participant, conducted at weekly intervals. At each visit, field workers collected saliva using a sterile oral swab. Swabs remained in the children's mouths for at least 20 seconds to ensure adequate absorption of saliva. Swabs were then placed into collection tubes and refrigerated during transport to the laboratory within 24 hours, where they were stored at -80°C pending laboratory analysis.

At each in-home visit the field workers also collected urine using a diaper insert. Filter papers (Whatman 903 grade, 1″ × 4″) were inserted into the inner panels of the diapers prior to putting them on the children. After urination, the inserts were removed from the diapers and air dried. Diaper inserts were transported to the laboratory within 24 hours, and stored at -80°C pending laboratory analysis.

During the last two weeks of follow up, the parents collected daily saliva and urine samples using the methods described above; these specimens were stored at 4°C (home refrigerator) until they were picked up by the field workers during their weekly visits. Among 864 possible specimen collection opportunities (36 children × 24 possible collection days), only 16 saliva specimens and 18 urine specimens were uncollected or were inadequate for testing.

### Laboratory testing

As described in more detail in Stowell et al. (companion paper), DNA was extracted from oral swabs through a quick extract method and from filter paper via thermal shock method [15]. In both cases, specimens were evaluated for the presence of CMV DNA using real-time PCR [16].

The limits of PCR detection were estimated to be 1,600 copies/mL for saliva and 16,000 copies/mL for urine. These limits are considerably higher than our detection limit for sterile specimens (e.g., blood) collected in clinical settings, which is 70 copies/mL [17]. Two factors led to these higher limits of detection: 1) To avoid false-positives, the PCR assay cutoff was raised five-fold from one copy per reaction (70 copies/ml) to five copies per reaction (350 copies/ ml) because saliva is not sterile and urine was collected in an unsterile manner, and unsterile specimens are more susceptible to trace amounts of contamination from the environment; 2) The methods of specimen collection (i.e., swabs and filter paper) were necessary to enable in-home collection by mothers, but they resulted in reduced sample volume which raised the limit of detection approximately five-fold for saliva in swabs and 50-fold for urine in filter paper.

### Statistical analysis

We used a Chi-square test to compare shedding prevalences, and linear regression to measure the association between age and log_10_ viral loads. We used generalized estimating equations that accounted for within-person correlation to compare median viral loads in saliva and urine, and to assess risk factors for shedding.

## Results

Of the 36 children, 20 (56%) were boys and 16 (44%) were girls; 20 (56%) were white, 12 (33%) were Asian-American, and 4 (11%) were of other race/ethnicity. At enrollment, 11 (31%) were less than 12 months of age, 12 (33%) were 12-23 months of age, and 13 (36%) were 24-47 months of age. Of the 32 mothers of these children (four mothers had two children in the study), 29 (91%) were CMV-seropositive.

**Figure 1:**
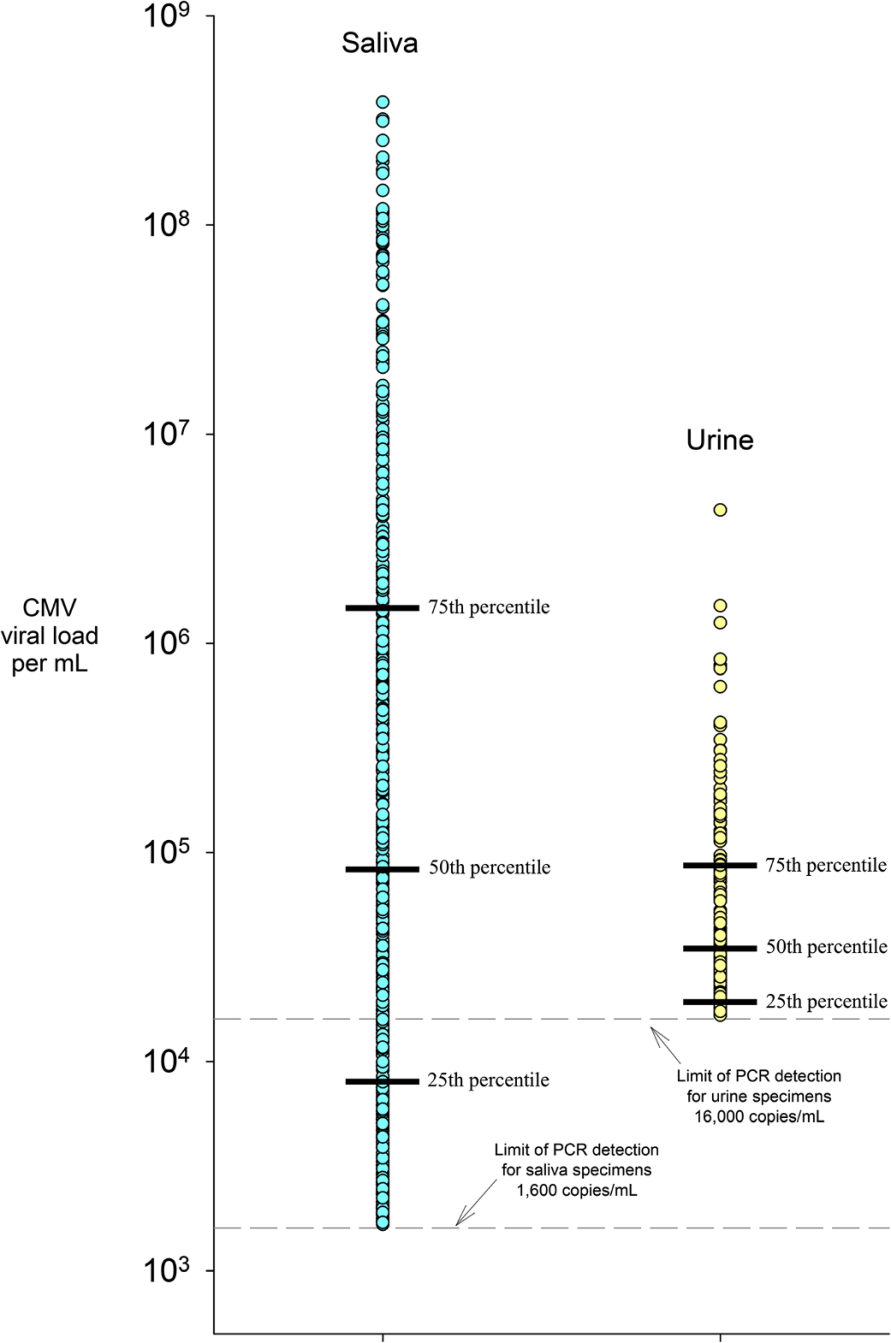
**Comparison of saliva and urine viral loads per mL across all specimens collected in longitudinal follow up.** Circles are only plotted for children who were shedding; negative results (i.e., viral loads below the limit of detection) are not plotted. Blue circles represent saliva results, and yellow circles represent urine results. Shedding prevalences and viral loads are not directly comparable because the two specimen types had different PCR limits of detection, due to their different collection formats.

During approximately 12 weeks of longitudinal follow up with the 36 children, CMV DNA was detected in 59% of saliva specimens (502/846) and 20% of urine specimens (167/844) (Figure 1). However, a direct comparison of shedding prevalences is inappropriate because the limit of PCR detection for saliva was lower than for urine. When using the higher limit (i.e., 16,000 copies/mL), the shedding prevalence in saliva was 40% (335/846) (i.e., 167 saliva specimens had viral loads between 1,600 and 16,000 copies/mL).

The prevalence of shedding in CMV-seropositive children was highly correlated with initial shedding status. Those who were shedding in saliva and/or urine at the screening visit had CMV DNA detected in 84% of follow-up saliva specimens (455/543) and 28% of follow-up urine specimens (151/539); those who were not shedding at the screening visit had CMV DNA detected in 16% of follow-up saliva specimens (47/303) and 5% of follow-up urine specimens (16/305). Those who were shedding at the screening visit were also more likely to have attended day care (P = 0.03), but had similar ages and IgG antibody titers as those who were not shedding at the screening visit (data not shown). IgG avidity was not significantly different for those who were initially shedding and not shedding (P = 0.13); however, antibody avidity was high for all eight of the non-shedders who had avidity data, suggesting that their infections were not recent.

We found that younger children in our study had a higher prevalence than older children of CMV shedding in saliva and urine after adjusting for within-person correlation, but the association was only statistically significant for urine (P = 0.01, Table 1). Children who ever attended daycare had a higher prevalence of shedding in saliva and urine, but the association was not statistically significant (Table 1).

**Table 1:** **Risk factors for CMV shedding (yes vs. no) adjusting for within-person correlation**

	Saliva			Urine		
Variable	Odds ratio (shedding)	95% CI	P value	Odds ratio (shedding)	95% CI	P value
Sex						
Girl	1		0.88	1		0.21
Boy	1.1	0.3-4.2		0.6	0.3-1.3	
Age (months)						
>24	1		0.12*	1		0.01
13-24	4.0	0.9-17.8		2.8	1.3-6.2	
0-12	3.9	1.0-14.8		7.3	3.2-16.8	
Daycare attendance						
Never	1		0.13	1		0.15
Ever	2.4	0.8-7.8		1.8	0.8-3.8	

*P for shedding prevalence being homogeneous across age categories.

Among DNA-positive specimens, we found median viral loads of 82,900 copies/mL in saliva and 34,730 copies/mL in urine (P = 0.01), while the viral loads for the 75th percentile were nearly 1.5 million copies/mL for saliva compared to 86,800 copies/mL for urine. If the saliva specimens had used the same limit of detection as urine (i.e., 16,000 copies/mL), their median viral load would have been 629,370 copies/mL rather than 82,900 copies/mL. Approximately 30% of saliva viral loads were greater than one million copies/mL, with some even topping 100 million copies/mL. Less than 2% of the urine viral loads were above one million copies/mL (Figure 1).

**Figure 2:**
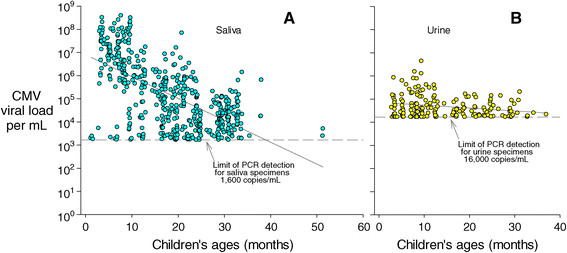
**CMV viral loads per mL as a function of children's ages in months.** Panel **A** shows results for saliva viral loads, and panel **B** shows results for urine viral loads. Circles are only plotted for children who were shedding; negative results (i.e., below the limit of detection) are not plotted. Blue circles represent saliva results, and yellow circles represent urine results. The regression line in Panel **A** is log_10_ (*CMV viral load*) =6.9-0.095 (*age in months*), with r^2^ = 0.39 and P < 0.001; the regression line in Panel **B** is log_10_ (*CMV viral load*) =4.9-0.012 (*age in months*), with r^2^ = 0.05 and P = 0.003.

At visits where children were shedding, log_10_ CMV viral loads were correlated with age (Figure 2). The correlation was especially strong for saliva: for example, children 12 months of age had average viral loads in saliva approximately 300 times those of children 36 months of age (P < 0.001). The correlation with urine was statistically significant (P = 0.003) but was not strong (regression coefficient = -0.012), nor did it explain much of the variance around the regression line (r^2^ = 0.05) (Figure 2).

**Figure 3:**
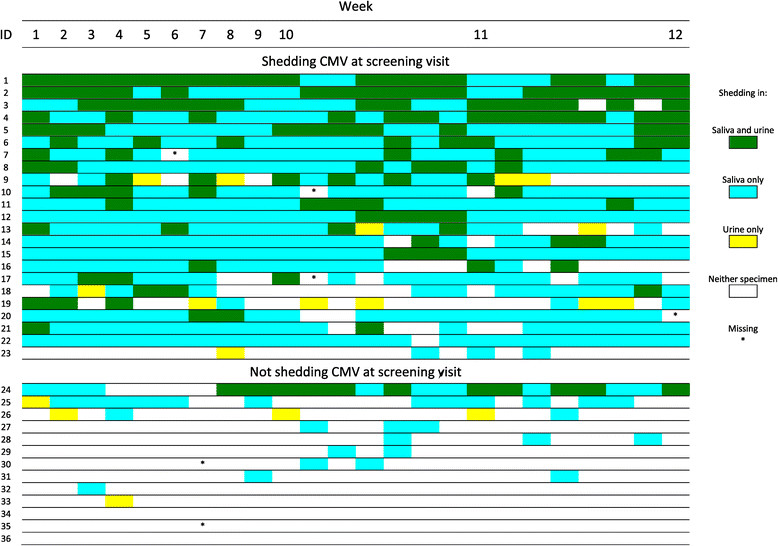
**CMV viral shedding among individual children by specimen type over the course of the study.**

All children who were shedding at the screening visit were still shedding on at least some of the days 10-12 weeks later, and the majority of these children were shedding in saliva and/or urine at nearly every data collection point (Figure 3). Among children who were not shedding at the screening visit, most shed at only a few visits during follow up (Figure 3). In general, children who were shedding in urine were also shedding in saliva, but children who were shedding CMV in saliva often were not shedding in urine. Some of this discrepancy may have been due to the less-sensitive PCR detection limit for urine. Nevertheless, children who were shedding in saliva were much more likely to also be shedding in urine than were children not shedding in saliva (unadjusted odds ratio = 7.7, p < 0.001). Similarly, children shedding in any fluid were much more likely to be shedding at the next visit compared to those who were not shedding (Table 2).

**Table 2:** **Correlation of CMV shedding from one visit to the next**

Shedding status by specimen:		*Positive* at current visit/ *positive* at previous visit (%)	*Positive* at current visit/ *negative* at previous visit (%)	Risk ratio	95% Confidence interval
Weekly visits	Urine	37/76 (49)	33/293 (11)	4.3	2.9-6.4
	Saliva	211/235 (90)	17/149 (11)	7.9	5.0-12.3
	Either specimen	218/245 (90)	18/132 (14)	6.5	4.2-10.1
Daily visits	Urine	47/91 (52)	44/354 (12)	4.2	3.0-5.9
	Saliva	229/267 (86)	24/174 (14)	6.2	4.3-9.1
	Either specimen	235/276 (85)	25/161 (16)	5.5	3.8-7.9

**Figure 4:**
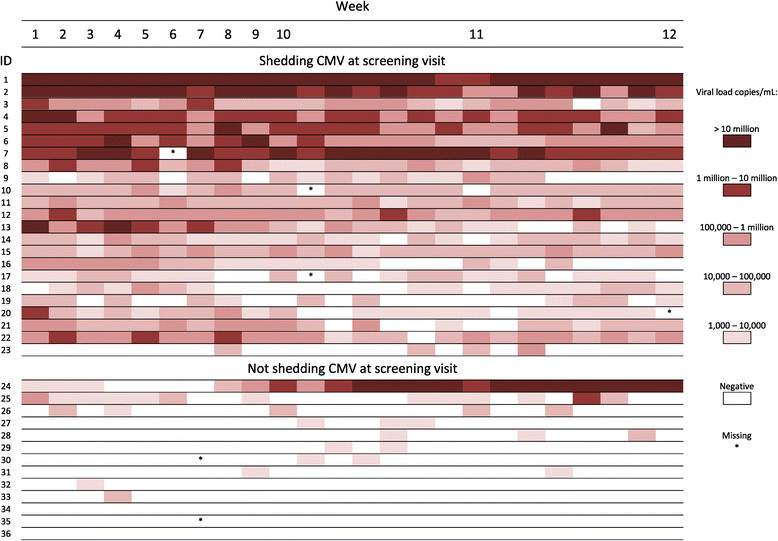
**CMV viral load among individual children over the course of the study.** Viral load was determined by taking the highest value of the saliva or urine viral load. Saliva viral load was highest in 801 of the 858 patient visits that had available results.

**Figure 5:**
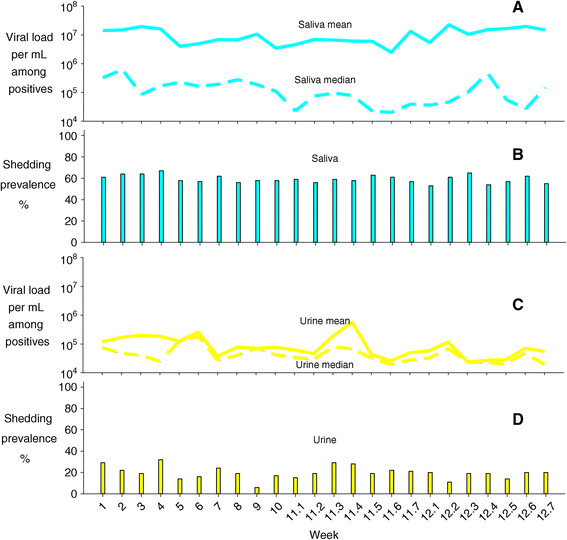
**CMV shedding prevalences and viral loads over time.** Panel **A**: Group mean and median CMV viral loads in saliva by visit number**.** Panel **B**: CMV shedding prevalences in saliva by visit number. Panel **C**: Group mean and median CMV viral loads in urine by visit number. Panel **D**: CMV shedding prevalences in urine by visit number.

**Figure 6:**
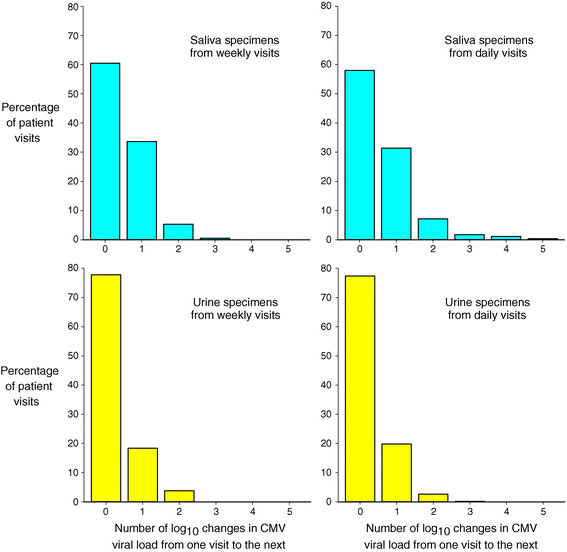
**Histogram of changes in CMV viral load category from one visit to the next for saliva and urine.** For example, a visit with a viral load in the 10^5^-10^6^ copies/mL category that was followed by a visit with a viral load in that same category would be added to the "zero" column of the histogram. On the other hand, if the visit was followed by a visit with a viral load in the 10^6^-10^7^ copies/mL category or the 10^4^-10^5^ copies/mL category, it would be added to the "one" column of the histogram.

Within individuals, CMV viral load varied somewhat over time during the follow-up period (Figure 4). However, the heaviest shedders at enrollment tended to continue to shed CMV at high levels. Children who were shedding in both body fluids tended to have higher viral loads (Figures 3 and 4). No major changes in either the group prevalences of shedding or the mean or median viral loads were observed during 12 weeks of follow up (Figure 5). Viral loads were also fairly stable at the individual level from one visit to the next (Figure 6).

## Discussion

Our main findings from this study were: 1) among healthy, young, CMV-seropositive children, those who were shedding CMV in saliva or urine tended to continue shedding for at least three months, usually at relatively stable levels; 2) a small subset of children shed at extremely high levels, and are presumably a more likely source of transmission to pregnant women; 3) the highest viral loads (>1×10^6^ copies/mL) were found in saliva; and 4) CMV shedding occurred at higher viral loads among younger children.

Some of the findings in this study were similar to previous observations, but several of our findings were novel. Many studies have examined CMV shedding among healthy children (reviewed in [18]), but few have collected longitudinal data [19]-[22]. We could not identify any studies that assessed CMV shedding at weekly or daily intervals as we did. In our study, we found that the presence of shedding was highly correlated over time and viral loads were relatively stable over days and weeks, even for as long as three months. This suggests that young children who are documented CMV shedders will probably continue shedding, and therefore may pose an ongoing risk to pregnant women. Our findings further suggest that even seropositive children who are initially not shedding have a reasonable chance of occasionally shedding CMV over the next few months, although usually at much lower viral loads (Figure 4). Our data suggest that children may typically shed CMV for a longer duration than adults, but it is difficult to make a valid comparison because longitudinal studies in adults have only assessed blood rather than saliva or urine [18]. If differences in viral loads or duration of shedding exist, they could be caused by physiological differences between children and adults (e.g., intensity of viral replication, immune system control), by a higher probability of recent infection among children, or by children's more frequent exposures to re-infection from other children. Differences in CMV viral loads have also been found in rhesus macaques, where juveniles had significantly higher viral loads than adults [23].

Another new finding was the extremely high viral loads in the saliva of healthy young children. Although a number of studies have measured CMV viral loads among children with congenital CMV infection [16],[24], few studies have measured viral loads among healthy children (i.e., not congenitally infected), the group most relevant for transmission to pregnant women. In fact, we were only able to identify two such studies [25],[26], and these were smaller than our study and reported only mean viral loads rather than the entire distribution of viral loads. Nearly half of the 502 CMV DNA-positive saliva specimens in our study contained >100,000 copies/mL and more than one quarter had >1×10^6^ copies/mL (Figure 1). In contrast, CMV viral loads in adults tend to be substantially lower (Stowell et al., companion paper) [27]. Many of the highest viral load specimens were obtained from a relatively small number of children (Figure 3)-perhaps 6 or 7 of the 161 originally screened. This may explain in part why CMV shedding is relatively common [18] and yet incident infections are relatively uncommon [14], i.e., the probability of transmission may be low unless viral loads are high.

The higher prevalence of shedding in saliva compared to urine was somewhat surprising. Most previous studies have found higher shedding prevalences in urine, typically 2%-20% higher than in saliva [18]. However, our large difference in shedding prevalence by specimen (40% in saliva vs. 20% in urine using the same limit of detection) cannot be compared to the results from those previous studies; our study design selected for shedders, including a preponderance of saliva shedders, and then sampled from them repeatedly. Our cross-sectional screening study, which is directly comparable to the previously published studies, showed an approximately 3% higher shedding prevalence in saliva, though the difference was not statistically significant (Stowell et al., companion paper). It is unclear why we did not find a higher shedding prevalence in urine, compared with saliva, as reported in previous studies. One possible explanation might be that our study and previous studies had important differences in laboratory procedures-we collected urine using filter paper and tested for CMV DNA using PCR, whereas all previous studies [18] collected liquid urine and tested for CMV using viral cultures.

On the other hand, the higher viral loads that we found in saliva compared to viral loads in urine were not necessarily unexpected. We identified two previous studies of healthy CMV seropositive children that compared viral loads in saliva to urine [25],[26]. In those studies, mean viral loads were about five times higher in saliva.

It is unclear why some seropositive children shed CMV while others did not. It is possible that a few of the younger children may have only had maternal IgG and no infection of their own. Four of the seropositive children who were not shedding at recruitment were younger than 12 months old. One of these children began shedding CMV at high levels later on, suggesting that at most three children could fall into the category of not being infected. For those who truly were infected, the occurrence of shedding may depend on the level of immune control, the particular viral strain, the time since initial infection, or less re-exposure to CMV reinfections.

Our study highlights the potential importance of saliva exposures for child-to-woman transmission of CMV. In addition to saliva sometimes having very high viral loads, women also report behaviors associated with potential exposures to children's saliva more frequently than behaviors associated with potential exposures to urine. According to a survey of more than 2000 women, kissing on the lips, sharing utensils, sharing cups, and sharing food are all common activities for women and children [28]. Women may also be exposed to saliva while wiping children's faces. In contrast, most exposures to urine occur while changing diapers, and most women report that they clean their hands after most diaper changes [28]. Importantly, many saliva exposures allow for direct transfer to mucous membranes, whereas most urine exposures require an intervening step involving hands, thereby reducing the chance for CMV to remain viable [29].

Another important conclusion from our study is that younger children may pose greater transmission risk than older children because of their tendency to shed CMV at higher levels. This is especially important since younger children also introduce more fluids into the environment (e.g., through drooling, mouthing toys, etc.) than do older children.

Thus, the framing and presentation of CMV prevention messages must take into account the potential risk posed by exposure to young children's saliva. In the past, some messages have emphasized hand washing, primarily as a way to reduce exposure to urine after diaper changing [5],[30],[31]. Although hand washing should remain an important part of prevention messaging, saliva exposures need to be addressed prominently. Intervention studies have shown that messages that highlight ways to reduce exposures to saliva are generally acceptable to women [6],[32].

Our study had several limitations. First, although we had more than 800 patient-visits and nearly 1,700 specimens tested, we followed a relatively small number of children (N = 36). It is possible that the children we followed differ in important ways from the general population of healthy, CMV-seropositive children. This might matter if, because of their age, race/ethnicity, or socioeconomic status, the children in our study were more or less likely than the general population of children to be re-exposed to CMV infection. The second limitation is that our study was not designed to assess risk factors for seroconversion, only risk factors for shedding. Although we could show that saliva can have extremely high levels of CMV DNA, we did not measure the extent to which such viral loads are associated with transmission to another person. A third limitation is that due to the conditions of specimen collection (e.g., in-home collection, filter paper collection, longer-term storage for the daily specimens) we did not routinely place the specimens in viral cultures but used PCR to quantify CMV DNA; thus, the presence of infectious virions was not demonstrated in most cases. This approach would be expected to overestimate the number of infectious particles in specimens. For a small number of specimens we also performed viral cultures and often found evidence for infectious virions but did not quantify them (unpublished data). However, the study conditions were chosen by design because they were the only way we could repeatedly collect specimens at short time intervals. A fourth limitation is that saliva and urine were collected using different materials (swabs vs. filter paper), which complicated between-fluid comparisons because urine had a higher limit of detection (Figure 1). Nevertheless, viral loads were substantially higher in saliva than in urine with or without adjustment for the different limits of detection.

## Conclusions

In conclusion, healthy, young, CMV-seropositive children can shed CMV for months at high, relatively stable levels. These data suggest that behavioral prevention messages need to address transmission via both saliva and urine, but also need to be informed by the potentially higher risks posed by saliva and by exposures to younger children.

## Authors' contributions

MJC conceived of the study, participated in its design, coordination, and data analysis, and drafted the manuscript. JDS helped design the study, coordinated and managed the data collection, and participated in data analysis. RC participated in data collection. PRD participated in data collection. DJ participated in data collection. KM participated in data collection. CS participated in data collection. KW participated in data collection. MA processed specimens and carried out laboratory testing. JG participated in the data analysis. DSS helped design the study. SCD helped design the study and supervised the laboratory testing. All authors read the manuscript, revised it critically for important intellectual content, and approved the final manuscript.

## Authors’ original submitted files for images

Below are the links to the authors’ original submitted files for images. Authors’ original file for figure 1 Authors’ original file for figure 2 Authors’ original file for figure 3 Authors’ original file for figure 4 Authors’ original file for figure 5 Authors’ original file for figure 6

## References

[1] Cannon MJ (2009). Congenital cytomegalovirus (CMV) epidemiology and awareness. J Clin Virol.

[2] Dollard SC, Grosse SD, Ross DS (2007). New estimates of the prevalence of neurological and sensory sequelae and mortality associated with congenital cytomegalovirus infection. Rev Med Virol.

[3] Mocarski ES, Shenk T, Pass RF, Knipe DM, Howley PM (2007). Cytomegaloviruses. Fields' Virology.

[4] Adler SP, Finney JW, Manganello AM, Best AM (1996). Prevention of child-to-mother transmission of cytomegalovirus by changing behaviors: a randomized controlled trial. Pediatr Infect Dis J.

[5] Cannon MJ, Davis KF: Washing our hands of the congenital cytomegalovirus disease epidemic. *BMC Public Health* 2005, 5(1):70., 10.1186/1471-2458-5-70 PMC1182379 15967030

[6] Vauloup-Fellous C, Picone O, Cordier A-G, Parent-du-Chatelet I, Senat M-V, Frydman R, Grangeot-Keros L (2009). Does hygiene counseling have an impact on the rate of CMV primary infection during pregnancy? Results of a 3-year prospective study in a French hospital. J Clin Virol.

[7] Pass RF, Zhang C, Evans A, Simpson T, Andrews W, Huang ML, Corey L, Hill J, Davis E, Flanigan C, Cloud G (2009). Vaccine prevention of maternal cytomegalovirus infection. N Engl J Med.

[8] Sung H, Schleiss MR (2010). Update on the current status of cytomegalovirus vaccines. Expert Rev Vaccines.

[9] Nigro G, Adler SP, La Torre R, Best AM (2005). Passive immunization during pregnancy for congenital cytomegalovirus infection. N Engl J Med.

[10] Johnson J, Anderson B, Pass RF (2012). Prevention of maternal and congenital cytomegalovirus infection. Clin Obstet Gynecol.

[11] Griffiths PD (2006). Progress towards interrupting intrauterine transmission of cytomegalovirus?. Rev Med Virol.

[12] Staras SA, Flanders WD, Dollard SC, Pass RF, McGowan JE, Cannon MJ (2008). Influence of sexual activity on cytomegalovirus seroprevalence in the United States, 1988-1994. Sex Transm Dis.

[13] Fowler KB, Pass RF (2006). Risk factors for congenital cytomegalovirus infection in the offspring of young women: exposure to young children and recent onset of sexual activity. Pediatrics.

[14] Hyde TB, Schmid DS, Cannon MJ (2010). Cytomegalovirus seroconversion rates and risk factors: implications for congenital CMV. Rev Med Virol.

[15] Barbi M, Binda S, Primache V, Caroppo S, Dido P, Guidotti P, Corbetta C, Melotti D (2000). Cytomegalovirus DNA detection in Guthrie cards: a powerful tool for diagnosing congenital infection. J Clin Virol.

[16] Boppana SB, Fowler KB, Pass RF, Rivera LB, Bradford RD, Lakeman FD, Britt WJ (2005). Congenital cytomegalovirus infection: association between virus burden in infancy and hearing loss. J Pediatr.

[17] Kharrazi M, Hyde T, Young S, Amin MM, Cannon MJ, Dollard SC (2010). Use of screening dried blood spots for estimation of prevalence, risk factors, and birth outcomes of congenital cytomegalovirus infection. J Pediatr.

[18] Cannon MJ, Hyde TB, Schmid DS (2011). Review of cytomegalovirus shedding in bodily fluids and relevance to congenital cytomegalovirus infection. Rev Med Virol.

[19] Murph JR, Bale JF (1988). The natural history of acquired cytomegalovirus infection among children in group day care. Am J Dis Child.

[20] Strom J (1979). A study of infections and illnesses in a day nursery based on inclusion-bearing cells in the urine and infectious agent in feces, urine and nasal secretion. Scand J Infect Dis.

[21] Levinsohn EM, Foy HM, Kenny GE, Wentworth BB, Grayston JT (1969). Isolation of cytomegalovirus from a cohort of 100 infants throughout the first year of life. Proc Soc Exp Biol Med.

[22] Stagno S, Pass RF, Dworsky ME, Alford CA (1983). Congenital and perinatal cytomegalovirus infections. Semin Perinatol.

[23] Antoine P, Varner V, Carville A, Connole M, Marchant A, Kaur A (2014). Postnatal acquisition of primary rhesus cytomegalovirus infection is associated with prolonged virus shedding and impaired CD4+ T lymphocyte function. J Infect Dis.

[24] Yan H, Koyano S, Inami Y, Yamamoto Y, Suzutani T, Mizuguchi M, Ushijima H, Kurane I, Inoue N (2008). Genetic variations in the gB, UL144 and UL149 genes of human cytomegalovirus strains collected from congenitally and postnatally infected Japanese children. Arch Virol.

[25] Murph JR, Bale JF, Murray JC, Stinski MF, Perlman S (1986). Cytomegalovirus transmission in a Midwest day care center: possible relationship to child care practices. J Pediatr.

[26] Bello C, Whittle H (1991). Cytomegalovirus infection in Gambian mothers and their babies. J Clin Pathol.

[27] Berntsson M, Dubicanac L, Tunback P, Ellstrom A, Lowhagen GB, Bergstrom T (2013). Frequent detection of cytomegalovirus and EpsteinBarr virus in cervical secretions from healthy young women. Acta Obstet Gynecol Scand.

[28] Cannon MJ, Westbrook K, Levis D, Schleiss MR, Thackeray R, Pass RF (2012). Awareness of and behaviors related to child-to-mother transmission of cytomegalovirus. Prev Med.

[29] Stowell JD, Forlin-Passoni D, Radford K, Bate SL, Dollard SC, Bialek SR, Cannon MJ, Schmid DS (2014). Cytomegalovirus survival and transferability and the effectiveness of common hand-washing agents against cytomegalovirus on live human hands. Appl Environ Microbiol.

[30] Red Book: 2012 Report of the Committee on Infectious Diseases. 2012, American Academy of Pediatrics, Elk Grove Village, IL, 29

[31] Perinatal Viral and Parasitic Infections. ACOG Practice Bulletin 20. 2000, ACOG, Washington, DC

[32] Adler SP (2011). Screening for cytomegalovirus during pregnancy. Infect Dis Obstet Gynecol.

