# Supplementary figures and images for: Repeated measures study of weekly and daily cytomegalovirus shedding patterns in saliva and urine of healthy cytomegalovirus-seropositive children
Source: BMC Infect Dis. 2014 Nov 13;14:569. doi: 10.1186/s12879-014-0569-1 (PMC4240830; doi:10.1186/s12879-014-0569-1)

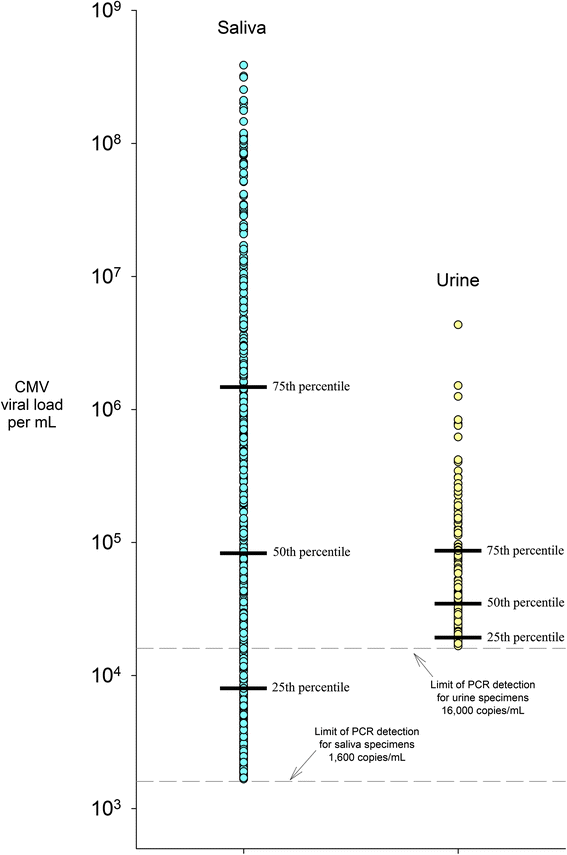

Supplement: Supplementary file 1 — Authors’ original file for figure 1 [file 12879_2014_569_MOESM1_ESM.gif]

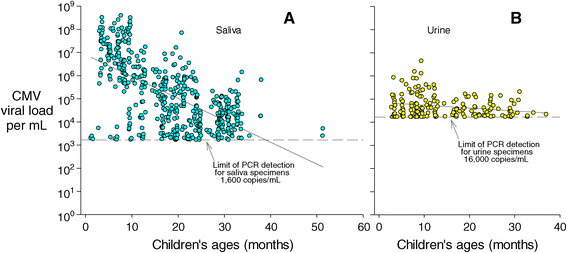

Supplement: Supplementary file 2 — Authors’ original file for figure 2 [file 12879_2014_569_MOESM2_ESM.gif]

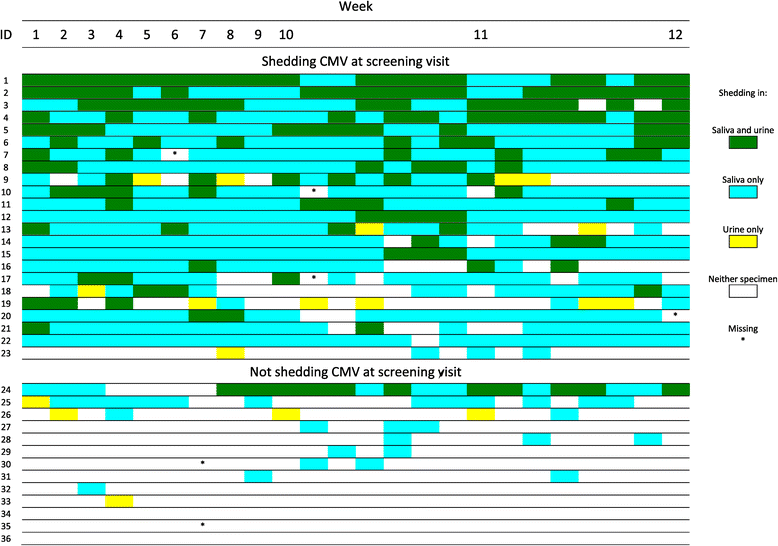

Supplement: Supplementary file 3 — Authors’ original file for figure 3 [file 12879_2014_569_MOESM3_ESM.gif]

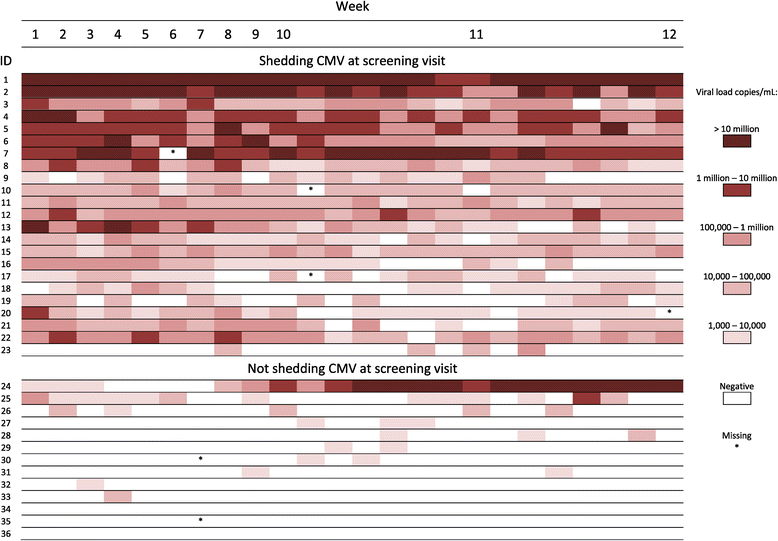

Supplement: Supplementary file 4 — Authors’ original file for figure 4 [file 12879_2014_569_MOESM4_ESM.gif]

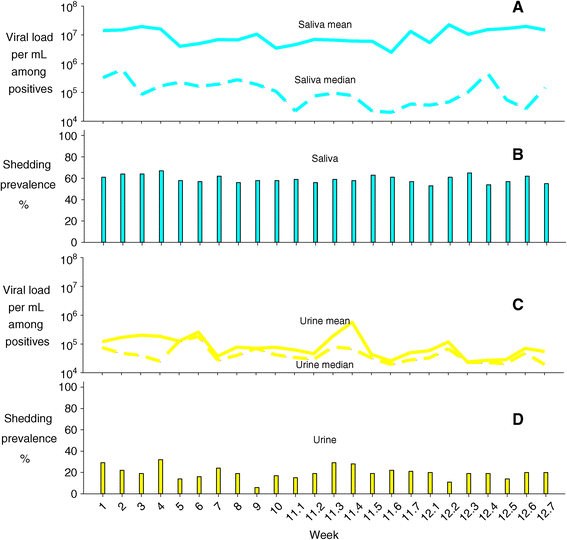

Supplement: Supplementary file 5 — Authors’ original file for figure 5 [file 12879_2014_569_MOESM5_ESM.gif]

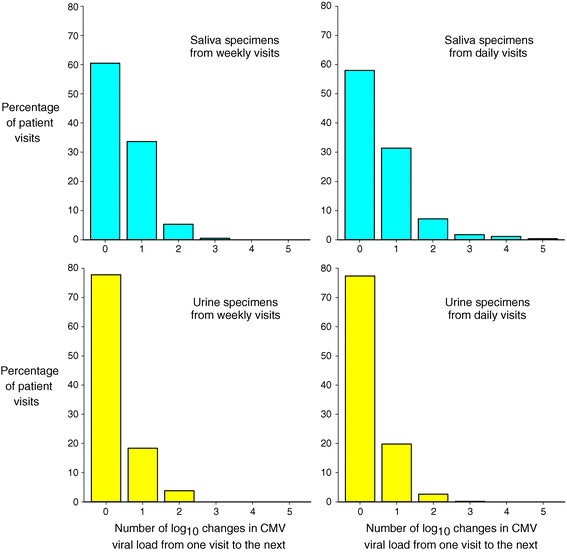

Supplement: Supplementary file 6 — Authors’ original file for figure 6 [file 12879_2014_569_MOESM6_ESM.gif]
